# Driving more WHO-recommended vaccines in the National Immunization Program: Issues and challenges in China

**DOI:** 10.1080/21645515.2023.2194190

**Published:** 2023-04-26

**Authors:** Peixi Dai, Qing Wang, Mengmeng Jia, Zhiwei Leng, Shuyun Xie, Luzhao Feng, Weizhong Yang

**Affiliations:** a Division of Infectious Diseases, Chinese Center for Disease Control and Prevention, Beijing, China; b School of Population Medicine and Public Health, Chinese Academy of Medical Sciences and Peking Union Medical College, Beijing, China; c Department of Immunization Program, Zhejiang Provincial Center for Disease Control and Prevention, Hangzhou, China

**Keywords:** National Immunization Program, vaccines, vaccine-preventable diseases, disease

## Abstract

WHO-recommended vaccines substantially prevent and control vaccine-preventable diseases (VPDs), but their inclusion differs among countries and regions. We reviewed the application for WHO-recommended vaccines in China and described the concerns and obstacles in driving the inclusion of more vaccines into China’s NIP, including immunization strategies, financial barriers, vaccination services, and behavioral and social supply-side and demand-side factors. China has made significant efforts, however, they may not be sufficient until the inclusion of more WHO-recommended vaccines in the National Immunization Program (NIP), ensuring that the vaccination encompasses the whole life course of individuals, establishment of more trustworthy vaccination finance and procurement, increasing vaccine development, optimizing vaccine demand forecasts, improving the accessibility and equity of vaccination services, capturing the key points of behavioral and social drivers of vaccination on the demand side, and establishing holistic prevention and control from a public health perspective.

## Introduction

In 1974, the World Health Assembly passed a resolution on the Expanded Program on Immunization (EPI) and called on the World Health Organization (WHO) member states to establish a National Immunization Program (NIP) to reduce the incidence of vaccine-preventable diseases (VPDs).^1^ NIP refers to the planned vaccination in the population using the vaccine varieties and immunization procedures determined by the government.^2^ However, NIP progress is different in countries around the world. Japan has issued the National Immunization Law in 1948 to recommend vaccination to prevent 12 VPDs. With the update of vaccines, current NIP in Japan has been extended to 12 vaccines to prevent 16 VPDs.^3^ The United Kingdom (UK) published its first national routine immunization schedules in 1961, which has been updated successively. To this day, UK’s NIP has included 17 vaccines, and has progressed from targeting children to covering the elderly, pregnant women or adults.^4,5^ In the early period after the founding of the People’s Republic of China (PRC), infectious diseases were a major threat to people’s health and well-being.^6,7^ The incidence of 11 surveillance VPDs in China was 1.7% in 1959.^8^ In 1978, China launched an NIP to control the spread of infectious diseases. This program focused on controlling six diseases using four vaccines (measles vaccine, oral polio vaccine, diphtheria, tetanus, BCG, and pertussis vaccine), and established a complete cold chain system for vaccine storage and transport. In China, the cost of vaccines included in NIP is borne by China’s central finance, the government provides NIP vaccines to citizens free of charge, and non-NIP vaccines are voluntarily administered and self-funded by citizens.^2^ By 1990, they had complete coverage of 1-year-old children at the county level. By 1995, the planned immunization rates of provinces, counties, and townships had reached 85%.^9^ Since 2001, vaccination has entered the immunization planning stage. After four rounds of gradual expansion of immunization planning, 16 vaccines were included in the NIP. Of these, 13 variants of vaccines prevent 12 diseases in children.^10^ Based on scientific research and standardization, immunization planning also comprised legislative changes to improve standardization and encourage evidence-based practices. Further, Healthy China 2030 was published in 2016 as an action program to promote the construction of a healthy China.^11^ The National Advisory Committee on Immunization (NACI) was established in 2017 to provide evidence-based support and technical vaccination expertise.^12^ In 2019, the Vaccine Administration Law of the PRC was passed to strictly regulate the entire process of vaccination, thereby ensuring the safety, efficacy, and accessibility of vaccines and increasing the public’s confidence in vaccination.^13^

With the expansion of the NIP over the past 40 years, several infectious diseases, including poliomyelitis and diphtheria, which pose serious threats to public health, have been controlled effectively.^11^ China’ NIP has contributed to a significant decrease in the incidence of several VPDs, such as pertussis, measles, epidemic cerebrospinal meningitis, and epidemic encephalitis B.^7,14^ For example, the incidence rate of measles decreased from 1.3% in 1965 to 4 in ten million in 2021; pertussis incidence rate decreased from 1965.6 per million in 1975 to 6.8 per million in 2021.^15^ There is evidence showing that annual VPDs mortality peaked at 499.6 per million in 1959, and the total number of VPDs deaths exceeding 335,700 cases. By 2017, mortality decreased by more than 99% to less than 0.38 per million.^8^

In recent decades, China’s NIP has significantly contributed to improving the response to VPDs. However, the Chinese public health needs for disease prevention exceeded the current coverage of NIP vaccines, and China faces more challenges to effectively control some serious VPDs, including pneumococcal diseases, influenza, cervical cancer, and herpes zoster. Most vaccines against these diseases are relatively new and are excluded from China’s NIP. Nevertheless, most of them have been approved for marketing in China and are recommended by the WHO. The difference in the types, coverage, and immunization strategies employed for such non-NIP vaccines across provinces and cities have hindered their use by the Chinese population. The WHO pointed out in the Immunization Agenda 2030, that a stronger national immunization infrastructure should be established and integrated into the primary healthcare service system, and that the healthcare system should be strengthened to ensure the effective implementation of immunization programs.^16^ Therefore, there is an urgent need to review the status of practical application of WHO-recommended vaccines in China, and for new proposals to help prevent and control VPDs effectively. In this study, we conduct a mini-review of Chinese vaccine inclusion challenges in terms of process, coverage, supply-side funding and facilitation strategies, and demand-side drivers, with targeted recommendations to provide considerations and improvement points for the development of a vaccine inclusion framework for NIP.

## Challenges of expanding China’s NIP

### Slow progress in expanding the coverage of China’s NIP

Evidence limited by native disease burden for further decision and action to include more vaccines in NIP, it is a slow progress in expanding the coverage of China’s NIP. In 2017, the WHO recommended countries to include the haemophilus influenza type B (Hib), pneumococcal conjugate (PCV), rotavirus, and human papillomavirus (HPV) vaccine in their national routine immunization schedules.^17^ Consequently, several developed and developing countries have gradually expanded the range of vaccines employed by their NIPs. However, relative to their international peers, China’s NIP is not completely inclusive of the WHO recommended vaccines that prevent some important VPDs (Table 1).

**Table 1.** Types of vaccines included in the NIP in selected countries.^14^.

Vaccines	China	The US	The UK	Japan	Russia	India	Kenya	Guinea	Sierra Leone
Hepatitis B	√	√	√	√	√	√	√	√	√
Bacillus Calmette – Guerin	√	-	√	√	√	√	√	√	√
Polio	√	√	√	√	√	√	√	√	√
Diphtheria, tetanus, and Pertussis	√	√	√	√	√	√	√	√	√
Measles	√	√	√	√	√	√	√	√	√
Rubella	√	√	√	√	√	√	√	-	√
Mumps	√	√	√	-	√	-	-	-	-
Japanese Encephalitis	√	√	-	√	-	√	-	-	-
Meningococcal	√	√	√	-	√	-	-	√	-
Hepatitis A	√	√	-	-	√	-	-	-	-
Rotavirus	-	√	√	-	-	√	√	√	√
Varicella	-	√	√	√	-	-	-	-	-
Hib	-	√	√	√	√	√	√	√	√
PCV	-	√	√	√	√	√	√	√	√
Influenza	-	√	√	√	√	-	√	-	-
HPV	-	√	√	√	√	-	√	√	√
Yellow fever	-	√	-	-	-	-	√	√	√

√: NIP vaccines.

-: Non-NIP vaccines.

The above vaccines are recommended by WHO to be included in NIP.

An unexpected factor weakens the process of vaccine inclusion as well. The emergence of Coronavirus Disease 2019 (COVID-19) interrupted routine immunization services: Vaccination clinics closed or opened fewer hours, while people was reluctant to seek health care for fear of infection or face the challenges in accessing immunization services due to blockades and transportation disruptions. Responses to the emerging infectious diseases led to business closures and worker job losses, as well as decreased productivity and economic recession, undoubtedly reducing the use of new vaccines that require out-of-pocket payments and affecting the original progress of vaccine inclusion even now that the economy is recovering.

### Lack of attention to the life course and high-risk population

WHO-recommended vaccines not included in the NIP in China (non-NIP vaccines) in China are offered primarily to children and older people, and are free for certain age or gender groups in some areas (Table 2). In rural areas, the non-NIP vaccine coverage is even lower than 50%.^26^ There is continued inadequate availability of vaccines targeted at adults, as in the case of the free influenza vaccines and the nine-valent HPV vaccines for males. Additionally, there is a bigger gap between the vaccination recommendations for adults and the at-risk population in China compared to that in developed countries. Immunization programs in developed countries, such as Germany,^27^ the US,^28^ and Australia,^29^ cover a life course of vaccine immunization, thereby maintaining a high immunization coverage and better control of VPDs. For example, in the US, the coverage of the herpes zoster vaccine for adults aged≥60 years was 34.5% in 2018.^30^ In contrast, a herpes zoster vaccine was only approved for marketing in China in 2020, and its current adoption is believed to be very low, although there is a lack of systematic studies examining this.

**Table 2.** Chinese non-NIP vaccines in partial provinces and cities.

Vaccines^a^	Provinces	Priority target populations
Influenza^18,19^	Beijing	≥60 years old with registered residence, primary and secondary school students and secondary professional school students
	Shenzhen	All primary and secondary school students in the city, ≥60 years old (including registered residence members and non- registered residence elderly who have joined the local health insurance)
	Zhejiang	≥70 years old registered residence members
	Hubei	Students in Xiangyang city and children over 3 years old in kindergartens
	Hunan	Primary and secondary school students and senior citizens ≥65 years old who are citizens of Kaifu District, Changsha City residents
	Tibet	≥60 years old in Chengguan district of Lhasa city
PCV/PPV^18–22^	Shandong	One dose of vaccination is free for school-age children with registered residence members in Weifang city (PCV)
	Beijing	Resident ID card or social security card of ≥65 years old
	Shanghai	≥60 years old registered residence members
	Shenzhen	≥60 years old registered residence members
	Zhejiang	≥60 years old in partial cities and counties
	Jiangsu	≥65 years old registered senior citizens in Wuxi, Suzhou, and Nantong city
HPV^23,24^ (15 pilot sites)	Fujian	Females 13–14 years old with semi-local school registration in Xiamen city
	Inner Mongolia	13–18 years old females in Junghar Banner, Erdos
	Shandong	Jinan City females age ≤14 years old in the seventh-grade
	Sichuan	13–14 years old females with local school registration in Chengdu city
	Guangdong	Females ≤14 years old who are in their first year of junior high school and have not received HPV vaccination with Guangdong school registration
Chickenpox^19,22,25^	Beijing	Children ≤15 years of age in nursery schools, primary and secondary schools where chickenpox outbreaks have occurred
	Shanghai	Children who are permanent residents of Shanghai and born on or after August 1, 2014
	Shenzhen	Children who are permanent residents of Shenzhen and born on or after December 1, 2014
	Jiangsu	Children residing in the cities of Nanjing, Wuxi, Suzhou, Changzhou, Yancheng, Zhenjiang, and Nantong cities

^a^The type of vaccine is included in the local immunization plan of partial provinces, cities, districts, and counties.

In addition to vaccines for adults, guidelines concerning the vaccination of professional groups, such as health workers (HWs), also include several issues. For example, HWs are considered a high-risk group for influenza infection, regular vaccination against seasonal strains of influenza is recommended in most European countries. Similarly, Australian influenza vaccination guidelines require immunization coverage among HWs to reach 95%, and New South Wales mandates those in clinically high-risk areas to be vaccinated.^31^ After Colombia introduced a policy in 2013 requiring medical personnel to be vaccinated against influenza, the coverage of this group reached 86%.^32^ Recently, China has also made improvements in this area. The National Influenza Prevention and Control Plan, established in 2018, emphasized that Chinese medical institutions should provide free influenza vaccines for HWs. Consequently, influenza vaccine coverage among HWs increased from 11.6% during the 2018/2019 influenza season to 67% during the 2019/2020 influenza season.^33,34^

### Centralized funding mechanism and high purchase price of non-NIP vaccines

The structure of vaccine financing, which is key to ensuring adequate quality and quantity of vaccines, is the basis for a fair and effective NIP. Developed countries, such as Japan,^35^ the US,^36^ the UK,^37^ and Germany,^27^ have diversified financing channels from central and local government finance, social medical insurance, private medical insurance, and individual payments, and make use of financial incentives to boost enthusiasm for vaccination. In China, NIP vaccines are purchased by the central government using tax revenue and non-NIP vaccines are purchased by the recipient.^38^ Therefore, the public’s incentive to vaccinate for expensive non-NIP vaccines, such as PCV and HPV, is significantly weaker.^39,40^ Some areas have included funding for partially non-NIP vaccines in their personal medical insurance plans and government financial subsidies. However, these measures benefit only a limited subset of the population (Table 2). Moreover, regional differences in the individual immunization costs contribute to higher vaccine coverage in economically developed regions and lower coverage elsewhere.

From the purchasing side, the Chinese zero-markup vaccine policy initiated in 2016 reduced the revenue of non-NIP vaccines, leading to the negativity of the purchaser. Greater demand for NIP vaccines in China has led to higher supply and lower procurement prices relative to the costs incurred by several high-income countries and the United Nations International Children’s Emergency Fund (UNICEF).^38^ However, lower purchase of non-NIP vaccines through the province’s Group Purchasing Organization system have resulted in underdeveloped supply chains supplying non-NIP vaccines at prices that are 5–20 times the UNICEF pricing.^41^ Additionally, provinces that rely on a single supplier for non-NIP vaccines can end up paying higher premiums. For example, in 2018, the price of PCV13 in Shanghai, which had only one source of supply, was approximately 1.7 times the average price in high-income countries. In contrast, the price of Hib and influenza vaccines, which are produced by multiple suppliers, is comparable to or lower than that incurred by the US, owing to market competition.^41^

### Over- and under-supply due to poor vaccine demand estimation

Inability of the healthcare providers and industry to accurately forecast the demand for non-NIP vaccines and adjust production accordingly has resulted in inadequate supply at times. Moreover, the vaccines are not supplied in time; for instance, the average delay is 90 days and above in 31 provincial-level regions and the Xinjiang Production and Construction Corps.^42^ In addition, there is a lack of research to provide timely estimations of the supply and demand of non-NIP vaccines. NIP vaccines are recommended for distinct target populations and, thus, has relatively stable annual demand, whereas non-NIP vaccines are purchased and used in response to fluctuating demand. Non-NIP vaccine shortages can occur because of unforeseen spikes in demand. In 2021, the annual production capacity of the coronavirus disease 2019 (COVID-19) vaccine in China exceeded seven billion doses, suggesting that failure to reasonably assess vaccine needs, rather than inadequate production technology capacity, results in imbalance between vaccine supply and demand. Several studies have been conducted on vaccine demand prediction methods worldwide.^43–45^ However, there is little research for methods of vaccine demand prediction specific to China, and most provinces estimate future demand based mainly on historical experience, resulting in imbalance between vaccine supply and demand.^46^ Furthermore, the supply shortage was further exacerbated by extension of vaccines manufacturing cycle for strict quality control, the lack of coordination between local CDC and manufacturers, and low incentives for manufacturers to increase supply.^47^ Imbalances between supply and demand directly affect immunization strategy implementation.

### Inter-regional disparities in service capacity

In terms of service capacity, evident regional differences exist in the number of vaccination units and workload of the inoculators. From 2004 to 2019, the cumulative number of vaccination units in China decreased by 72%, and the average number of inoculators nationwide was 1.7 serving 10,000 residents.^48^ The eastern China mostly had 29% of vaccination units with a daily workload of more than 30 doses per inoculator.^49^ The eastern region has relatively high living standards and vaccine awareness, and a dense and highly mobile population. Thus, the high and increasing demand for public vaccination services may strain the manpower available for immunization services.^46^ In remote areas with less developed transportation systems, workers must visit households to provide mobile services. In such cases, the vaccination unit does not have a clear geographical area of responsibility and the service radius of the vaccination sites is very large. Particularly, the number of township vaccination units with a service radius of more than 10 km in the west is twice than that in the east. The service radius of urban vaccination units also varies significantly between provinces. For example, the average service radius in Tibet is 59 km.^49^

Inter-regional differences are also reflected in standardized vaccination procedures. China has required in the Vaccines Administration Law establish a digital system for standardized vaccination procedures: use five separate digital identifiers – vaccine source, vaccine product, cold chain equipment, vaccinated child, and vaccinator to trace every dose of vaccine.^50^ However, China has generally achieved standardization only in urban regions and wealthy rural areas. Due to the constraints of resources and the low capacity of vaccinators, vaccination procedures such as vaccine storage, registration management, and vaccination observation in western rural areas are not standardized. In addition, the immunization information systems had cross-province record duplication, the within-province duplicate-record rate varied from 0.3% to 4.0%.^51^

### Weak demand-side drivers of vaccination

Driving more WHO-recommended vaccines for greater use in addition to supply-side considerations, also taking into account demand-side influences. The WHO proposed the Behavioral and Social Drivers of Vaccination (BeSD) for vaccination promotion in May 2022, to help stakeholders understand and identify the causes of vaccine underutilization and guide the identification, implementation, monitoring, and evaluation of interventions.^52^ In China, vaccines, such as the influenza vaccine, that were included in the regional routine immunization programs were administered to less than 10% of the general population. On the demand side of vaccination, the public is generally hesitant to vaccinate due to misconceptions about vaccine quality, safety, and efficacy, which are the main factors of weak drivers. Research-based evidence showed that 61.96% of parents hesitated to vaccinate with non-NIP vaccines and 17.5% were highly hesitant to vaccinate with non-NIP vaccines.^53^ Parents consider vaccination to be highly risky, thus, resulting in public distrust and a decreased rate of non-NIP vaccination.^54^ The infodemic and the low drive to vaccinate are mutually reinforcing – misinformation or disinformation weakens the willingness to vaccinate, which further leads to the next information pandemic and repeats itself.^55^ In a study of low- and middle-income countries, perceived vaccine safety and effectiveness were the two most important contributors to the intention to receive an influenza vaccine.^56^ Furthermore, people do not fully understand the harm of the pandemic and the importance of vaccination, which also leads to a gap between willingness to vaccinate and actual action, and ultimately to the abandonment of vaccination.^57^ Hence, the aforementioned main weak demand-side drivers indirectly and negatively affect the priority of decision makers regarding the inclusion of WHO-recommended vaccines in the NIP.

## Reflections and suggestions

### NIP expansion and adoption a whole life-course approach

China must develop a theoretical framework for the inclusion of new vaccines in NIP. The new vaccines can be successfully introduced and streamlined in the immunization program with good program leadership and planning, and these experiences could be extended to other health interventions.^58^ The factors considered by other countries can also be referenced during this process. For example, the Netherlands adopted seven selection criteria for deciding on candidate vaccines for inclusion in its NIP: seriousness and extent of the disease burden, effectiveness, safety, acceptability, efficiency, and priority.^59^ Notably, most VPDs, such as pneumonia, are not notifiable infectious diseases in China, and have relatively fragmented surveillance data. Therefore, a robust surveillance and reporting system for VPDs and a multichannel early warning system with multipoint triggers are crucial for deciding the inclusion of vaccines in the NIP, and these constitute the evidence basis for the above evaluation study to be included in the NIP.

In addition, the WHO Immunization Agenda 2030 aims to attain whole life-course immunization services by 2030 and alleviate the burden of VPDs across the entire lifespan.^16^ To this end, China should expand the scope of target populations for immunization to encompass not only high-risk groups and school-age children, but also healthy and older adults, in order to provide greater protection and bridge the immunization gap.

### More trustworthy vaccination finance and procurement

Equity, efficiency, resource accessibility, and procedural rationality of specific vaccines should be considered for multichannel financing and economic risk sharing. The variable proportions of individual out-of-pocket payments, health insurance, commercial insurance, and central or local government payments should be reviewed regularly to reduce the financial burden on both the government and population. If the contribution of health insurance to vaccine financing increases, it could be further leveraged as a major source of funding, independent of government taxation and personal co-payments. During the COVID-19 pandemic, health insurance funding and local finances shared the cost of SARS-CoV vaccination, which provides a model for optimizing the NIP financing model. Regarding procurement, inspiration can be drawn from established and effective UNICEF procedures, with a focus on utilizing large-scale collective bidding and price shifting, and the establishment of a tiered pricing scheme in which low-income areas benefit from lower vaccine prices. The success of the Global Alliance for Vaccines and Immunization in achieving cost-efficient global procurement should be studied and emulated.^60^

### Increasing vaccine development and optimizing vaccine demand forecasts

Domestic Chinese vaccine manufacturers are responsible for most of the supply of NIP vaccines in China, however, several companies pay limited attention to the research and development (R&D) of novel vaccines.^61^ Therefore, it is necessary to actively engage in interdepartmental cooperation in non-NIP vaccine R&D, production, and distribution. Health authorities should estimate the demand for vaccines by analyzing specific local factors that affect adoption, and cooperate with vaccine producers to encourage R&D in appropriate areas, with an emphasis on reducing periods of excessive or insufficient production.

### Improving the accessibility and equity of vaccination services and formulating more supportive policies

Better functioning of the NACI needs to be improved to update supportive vaccine programs based on updated evidence or local disease characteristics. This relies on the joint efforts of different ministries, such as the State Council, the State Drug Administration, the Ministry of Finance, the National Health Insurance Agency, and the National Health Commission, to periodically develop a feasible work plan. Regarding the factors affecting non-NIP vaccination in different regions, the policies proposed by public health policymakers should be in line with the local realities. In developing areas with low public awareness, supporting health workers’ knowledge dissemination and free vaccination should be prioritized; and mandatory vaccination will help them recommend it to patients. In developed regions, non-NIP vaccine policies should be more inclined toward post-vaccination evidence collection, such as adverse reaction monitoring, to facilitate faster incorporation into routine immunization.

Professional vaccine inoculators and service systems should be optimized according to the health needs and challenges of the public. Healthcare authorities are responsible for coordinating vaccine introduction, distribution, and management. This should involve the provision of demand-mapping resources based on both resident and mobile populations, as well as the establishment of vaccination units and enhancement of workforce planning to optimize the allocation of prophylactic inoculation medical care personnels. Furthermore, multiple vaccination service patterns for adults, similar to the integration of vaccination clinic programs with the family doctor-contracting system, have been explored to provide quality services. Furthermore, makeshift vaccination sites and mobile vaccination vans can be established in remote areas for better accessibility and equity, thereby maximizing the health benefits for the whole population.

### Capturing the key points of BeSD on the demand side

Leveraging demand-side drivers to provide policymakers with more real-world evidence is one method for the inclusion of WHO-recommended vaccines. These drivers include consideration of the interpersonal and social environment of the vaccinating individual, social norms that encourage vaccination with the support of family or religious leaders, positive vaccination advice from healthcare workers, and gender-equitable vaccination. No single intervention strategy can address the complexity of all vaccine hesitancy to drive vaccination. Therefore, conducting area-based surveys to identify issues of hesitancy and targeted advocacy measures based on empirical data collected. Popularizing the knowledge of non-NIP vaccines through multi-channels, including regular vaccine-knowledge lectures and health education columns, would improve public awareness of VPD burden, vaccines safety, and effectiveness.^57,62^ The media monitors misinformation and false information that undermines confidence in vaccination and reduces the vaccination rate, and deals with it by “presenting the facts – warning of misinformation – explaining the fallacies – publishing the correct information.”^63^ Simultaneously, doctors should persistently recommend non-NIP vaccines. The doctor’s recommendation can significantly change people’s attitude toward vaccination, thereby inducing a positive belief in the effectiveness and safety of non-NIP vaccines.^64^

### The COVID-19 vaccines campaign insights relevant to non-NIP vaccinations

Between March 2021 and May 2022, 88% of mainland China’s population completed the full schedule of COVID-19 vaccination. This can be attributed to the coordination between different organizations, including local, regional, and national healthcare bodies, and individual hospitals. To encourage the adoption of non-NIP vaccines, multi-departmental collaboration among hospitals, public health authorities, and communities should be encouraged to improve access to rural and temporary vaccination sites. Schools, kindergartens, nursing homes, and other organizations can also be involved in centralized vaccination efforts to facilitate door-to-door vaccination. In addition, public institutions, communities, and enterprises can negotiate with vaccination units to encourage vaccination among their employees or residents.

## Conclusions

Vaccination is the most cost-effective method for the prevention and control of VPDs. Despite advances over the past few decades, a gap still exists between China and developed and some developing countries, regarding immunization strategies, disease surveys and reporting, primary healthcare service systems, and the production and distribution capacity of non-NIP vaccines. Proactive strategies and measures for surveying and evaluating VPDs, and financing, procurement, vaccination services, and warranties and policy support for relevant non-NIP vaccines, have been established. These contribute to the better prevention and control of VPDs and the access of the Healthy China Initiative.
